# Hitching a Ride in the Phyllosphere: Surfactant Production of *Pseudomonas* spp. Causes Co-swarming of *Pantoea eucalypti* 299R

**DOI:** 10.1007/s00248-024-02381-4

**Published:** 2024-04-29

**Authors:** Michael Kunzler, Rudolf O. Schlechter, Lukas Schreiber, Mitja N. P. Remus-Emsermann

**Affiliations:** 1 Institute for Biology - Microbiology, Freie Universität Berlin, Königin-Luise Straße 12-16, 14195 Berlin, Germany; 2 Institute for Cellular and Molecular Botany, Bonn University, Kirschallee 1-3, 53115 Bonn, Germany

**Keywords:** Fluorescent proteins, Bioreporter, Reproductive success, CUSPER, Bacteria-bacteria interactions

## Abstract

Here, we demonstrate the beneficial effect of surfactant-producing pseudomonads on *Pantoea eucalypti* 299R. We conducted a series of experiments in environments of increasing complexity. *P. eucalypti* 299R (Pe299R), and *Pseudomonas* sp. FF1 (Pff1) or Pe299R and surfactant-production deficient *Pseudomonas* sp. FF1::ΔviscB (Pff1ΔviscB) were co-inoculated in broth, on swarming agar plates, and on plants. In broth, there were no differences in the growth dynamics of Pe299R when growing in the presence of Pff1 or Pff1ΔviscB. By contrast, on swarming agar plates, Pe299R was able to co-swarm with Pff1 which led to a significant increase in Pe299R biomass compared to Pe299R growing with Pff1ΔviscB or in monoculture. Finally in planta, and using the single-cell bioreporter for reproductive success (CUSPER), we found a temporally distinct beneficial effect of Pff1 on co-inoculated Pe299R subpopulations that did not occur in the presence of Pff1ΔviscB. We tested three additional surfactant-producing pseudomonads and their respective surfactant knockout mutants on PE299R on swarming agar showing similar results. This led us to propose a model for the positive effect of surfactant production during leaf colonization. Our results indicate that co-motility might be common during leaf colonization and adds yet another facet to the already manyfold roles of surfactants.

**Supplementary Information:**

The online version contains supplementary material available at 10.1007/s00248-024-02381-4.

## Introduction

The microbial habitat that is presented by leaf surfaces, the phyllosphere, is densely colonized by microorganisms. Among these microorganisms, bacteria are the most abundant and prevalent group, establishing non-random patterns of colonization on leaves. Bacteria have a tendency to aggregate with one another in close proximity, rather than being distributed homogeneously along the leaf surface, suggesting the role of deterministic processes on leaf colonization [1].

In recent years, much attention has been paid to leaf colonizers and the factors that drive leaf colonization at the population and the community level. For example, the host plant species influences leaf colonization and bacterial community composition [2–4], leading to recurring patterns of bacterial communities on leaves, at least at low phylogenetic resolution, from year to year [5, 6].

At the same time, microbe-microbe interactions affect community assemblages on leaves [7–9]. However, many of the mechanisms driving these interactions remain unclear. In studies where bacterial communities are relatively simple, it appears that overlap in resource utilization ability of competitors has a limited impact on leaves [8, 10, 11]. This suggests that the highly segregated leaf environment constrains interspecies bacterial interactions, despite their tendency to co-aggregate [12, 13]. As proximity is a key factor for interaction, mobility might emerge as an important factor that is shaping interactions and, ultimately, bacterial communities. In general, mobility on leaves has received limited attention, with most studies being related to the presence of flagellar genes in *Pseudomonas* spp. [14–16]. Since roughly 5% of the leaf surface is colonized by bacteria [13] and not all leaf-colonizing bacteria are flagellated [17], other means of mobility must be present on leaves. This may include movement mediated by colony expansion or gliding motility [18, 19], or spread by dew [20, 21]. The genera *Pantoea* and *Pseudomonas* are known to be very common leaf colonizers [6, 22]. Both taxa interact with their plant host in various ways, ranging from pathogenicity to plant protection [23–26]. However, their interaction within communities has not received much attention, despite some initial efforts [12].

Phyllosphere-colonizing pseudomonads have previously been studied with regard to their ability to produce surfactants [27–31]. Interestingly, a fitness advantage of surfactant-producing pseudomonads over their surfactant-deficient counterparts was evident only under specific conditions such as fluctuating humidity [27]. Otherwise, surfactant production did not seem to affect the ability of pseudomonads to colonize leaves [29].

Surfactants have been suggested to have multiple roles in the phyllosphere, including increased spread of water by reducing surface tension, improved nutrient diffusion from the apoplast to the phyllosphere by increasing cuticle permeability [31], and increased drought resistance due to their hygroscopic nature [27, 28]. On semi-solid media, surfactants are necessary for swarming, and it has been shown that they can facilitate the mobilization of some bacteria in a process called co-swarming [32]. However, the effect of surfactant-producing bacteria on a second colonizer in the phyllosphere has, to our knowledge, not been studied.

Bacterial mobility relies on various mechanisms. Swimming motility is an active form of movement, powered by rotating flagella, occurring in liquid or low-viscosity conditions [33]. Gliding motility is movement on surfaces, independent of flagella or pili [34]. Another surface-associated motility mode is sliding motility, driven by excreted biosurfactants, preventing cells from forming thick biofilm layers and aiding dispersal on the surface they inhabit [35]. Twitching motility is mediated by type IV pili, which propels bacteria by retraction, while swarming motility is the coordinated movement of multiple bacteria across solid or semi-solid surfaces, requiring flagella, a functional quorum sensing system, and surfactant biosynthesis [36]. With this mechanism, bacteria co-migrate in side-by-side groups called rafts, instead of individually as in swimming motility [37].

Previously, we developed a bioreporter, CUSPER, to measure the number of divisions of individual cells of the bacterium *Pantoea eucalypti* 299R (formerly known as *Erwinia herbicola* 299R and *Pantoea agglomerans* 299R) after they arrive in new environments. This bioreporter for reproductive success is based on the dilution of green fluorescent protein (GFP) during cell division. Thereby, the GFP intensity becomes a direct proxy for the number of experienced divisions [38, 39]. In a previous study, we observed that *Pseudomonas* spp. increased the single-cell reproductive success of *P. eucalypti* 299R (Pe299R) in planta despite being strong competitors in vitro [11]. Given the common feature of surfactant production in pseudomonads [30, 40], we hypothesized that surfactant production increases the reproductive success of a second colonizer in the phyllosphere. To test this, we investigated the interactions between Pe299R, surfactant-producing *Pseudomonas* isolates, and their respective knockout mutants in various conditions, ranging from liquid medium, agar surfaces, and leaves. We observed contrasting results in liquid cultures compared to agar surfaces and leaves.

## Material and Methods

### Bacteria, Strain Construction, and Growth Conditions

All bacteria used in this study are listed in Table 1. Bacteria were routinely grown on lysogeny broth agar (LB-Agar, HiMedia). *Pantoea eucalypti* 299R was equipped with a constitutively expressed red fluorescent mScarlet-I protein gene and the plasmid pCUSPER [11]. The resulting strain will be referred to as Pe299R_CUSPER_ from here onwards. To maintain the plasmid carrying the reproductive success construct in Pe299R_CUSPER_, the agar was supplemented with 50 mg L^−1^ kanamycin (Roth). Constitutively cyan-fluorescent derivatives of strains *Pseudomonas* sp. Pff1 (Pff1_cyan_) and *Pseudomonas* sp. Pff1::ezTn5-viscB (Pff1ΔviscB_cyan_) used in this study were generated using plasmid pMRE-Tn7-141 [41] explained elsewhere using conjugation and the auxotrophic donor strain *E. coli* ST18 [42, 43]. Strains *Pseudomonas* sp. Pff2, *Pseudomonas* sp. Pff2::ezTn5-viscB, *Pseudomonas* sp. Pff3, *Pseudomonas* sp. Pff3::ezTn5-massB, *Pseudomonas* sp. Pff4, and *Pseudomonas* sp. Pff4::ezTn5-massB were characterized elsewhere and carry Tn*5-*knockout insertions in their respective surfactant production genes: viscosin B (*viscB*) or the massetolide B (*massB*) genes [29]. Those genes were previously shown to be responsible for surfactant production.

**Table 1:** Bacterial strains used in this study

Name	Relevant genotype/ properties	Antibiotic resistance	Abbreviation	Origin
*Escherichia coli* ST18	pMRE-Tn7-145; red fluorescent	Cm, Gent, Amp		[41]
*Escherichia coli* ST18	pMRE-Tn7-141; cyan fluorescent	Cm, Gent, Amp		[41]
*Pantoea eucalypti* 299R		Rif		[44]
*Pantoea eucalypti* 299R	::Tn7-145; pCUSPER; red fluorescent, growth bioreporter	Rif, Gent, Kan	Pe299R_CUSPER_	[11]
*Pseudomonas* sp. FF1	Produces viscosin B		Pff1	[29]
*Pseudomonas* sp. FF1	::Tn7-141; cyan fluorescent	Gent	Pff1_cyan_	This study
*Pseudomonas* sp. FF1	::ezTn5-viscB	Kan	Pff1ΔviscB	[29]
*Pseudomonas* sp. FF1	::ezTn5-viscB::Tn7-141; cyan fluorescent	Gent, Kan	Pff1ΔviscB_cyan_	This study
*Pseudomonas* sp. FF2	Produces viscosin B		Pff2	[29]
*Pseudomonas* sp. FF2	::ezTn5-viscB	Kan	Pff2ΔviscB	[29]
*Pseudomonas* sp. FF3	Produces massetolide B		Pff3	[29]
*Pseudomonas* sp. FF3	::ezTn5-massB	Kan	Pff3ΔmassB	[29]
*Pseudomonas* sp. FF4	Produces massetolide B		Pff4	[29]
*Pseudomonas* sp. FF4	::ezTn5-massB	Kan	Pff4ΔmassB	[29]

### In Vitro Co-inoculation Assay

To investigate the co-colonization of Pe299R_CUSPER_, Pff1_cyan_ or Pff1ΔviscB_cyan_ in vitro, all strains were grown in 3 mL M9 minimal media (Na_2_HPO_4_•7H_2_O 64 g L^−1^, KH_2_PO_4_ 15 g L^−1^, NaCl 2.5 g L^−1^, NH_4_Cl 5.0 g L^−1^) supplemented with 200 µM FeCl_3_ (to avoid production of autofluorescent pyoverdines by pseudomonads) and 0.13% glucose, fructose, and sorbitol each (M9 3C) overnight at 30 °C and 200 rpm. Five hundred microliters of each overnight culture were then used to inoculate 50 mL M9 3C, respectively. After 6 h, cultures were washed twice by centrifugation at 2000 *g* for 5 min and resuspended in PBS. Suspensions were diluted to an optical density (OD600nm) of 1 and the following treatments were prepared: Pe299R_CUSPER_, Pe299R_CUSPER_ vs. Pff1_cyan_, Pe299R_CUSPER_ vs. Pff1ΔviscB_cyan_, Pff1_cyan_, and Pff1ΔviscB_cyan_. These suspensions were diluted into either M9 supplemented with glucose 0.4% (M9gluc), M9 3C, or LB to a final OD600nm of 0.04 for each strain. Despite the lack of kanamycin, we did not expect any significant loss of plasmid during this experiment as the plasmid backbone of pCUSPER was described to be highly stable in Pe299R [45]. Furthermore, the fitness effects of near identical plasmids in Pe299R were described to be negligible [46]. Triplicate treatments were pipetted into a 96-well plate and placed into a CLARIOstar Plus microplate reader (BMG Labtech). The fluorescence of the mScarlet-I expressing Pe299R_CUSPER_ was determined by excitation at 530–570 nm and measuring emission at 580–620 nm, while mTurquoise2, produced by pseudomonads, was excited at 400–440 nm and its emission was measured at 450–490 nm using the CLARIOstar software (version 5.70, BMG Labtech). Measurements were performed in 20-min intervals for 42 h. The plate was incubated at 30 °C, and the plate was shaken in a double orbital at 200 rpm between measurements.

The area under the curve of the red and cyan fluorescence kinetics of each sample was determined in Prism 10.0.1 (Graphpad). As cyan fluorescence kinetics exhibited a negative change in fluorescence that was media dependent, the data was corrected by a constant value as to move every datapoint above the baseline before the absolute area under the curve was determined.

### Swarming Assays

To determine the swarming ability of Pe299R_CUSPER_, Pff1_cyan_, and Pff1ΔviscB_cyan_, each strain was inoculated onto soft agar. KB-based soft agar plates were prepared with 1.26% w/v King’s medium B, (LB-Agar, HiMedia) and 0.4% agarose. The center of the plate was inoculated with 10 µL of bacterial suspension (OD600nm = 1, prepared as above), and pictures were taken after 24 h of incubation at 30 °C using a dark field illumination stage [47] in a dark chamber (MultiImage Light Cabinet) with attached Axiocam 105 (Zeiss) and Zen Core (version 3.2, Zeiss).

LB-based soft agar was prepared by diluting one part LB agar with two parts ddH_2_O and supplementing with 200 µM FeCl_3_. Two mL soft agar per well was then distributed into a 6-well microtiter plate (Greiner). After the agar was gelled, a 1.5 µL drop of washed bacterial suspensions (adjusted OD600nm = 0.5) was placed in the middle of each well. The following monocultures and mixtures were prepared: (i) Pe299R_CUSPER_, (ii) Pe299R_CUSPER_ vs. Pff1_cyan_, (iii) Pe299R_CUSPER_ vs. Pff1ΔviscB_cyan_, and (iv) Pff1_cyan_, and Pff1ΔviscB_cyan_. The plates were then incubated at 30 °C for 16 h. Afterwards, the plates were placed into a CLARIOstar Plus Microplate reader (BMG Labtech). The fluorescence of the mScarlet-I expressing Pe299R_CUSPER_ was determined by exciting the sample at 530–570 nm and measuring emission at 580–620 nm while mTurquoise-producing pseudomonads were excited at 400–440 nm and its emission was measured at 450–490 nm. The plates were scanned using the 30 × 30 Matrix scan mode of the CLARIOstar software using bottom optics to obtain the spatial information of each strain. This resulted in a two-dimensional distribution of red and cyan fluorescence data. Background subtraction was performed individually for each datapoint. Data was visualized using the heatmap function of Prism. Total fluorescence of the red fluorescence channel was used as a proxy to determine the total biomass of Pe299R_CUSPER_. The experiment was performed four times on LB soft agar.

Additionally, to test if co-swarming also occurs when Pe299R_CUSPER_ co-colonizes agar surfaces with other swarming pseudomonads, Pe299R_CUSPER_ was co-inoculated with Pff2, Pff3, and Pff4, as well as their respective surfactant-deficient mutants on soft LB agar.

### Plant Growth

Plants were prepared as explained in Miebach et al. [48]. Briefly, *Arabidopsis thaliana* Col-0 seeds were surface sterilized by vortexing in 70% v/v ethanol for 2 min. The ethanol was then removed by pipetting, and the seeds were treated with 50% v/v household bleach (NaOCl, 2.47%) and 0.02% v/v Tween-20 for 7 min. Afterwards, the seeds were washed three times with sterile water. Sterilized seeds were kept in water and stratified in the dark at 4 °C for 3 days. After stratification, the seeds were sown on cut pipette tips (∼5 mm in length) pre-filled with ½ strength Murashige-Skoog (½ MS) agar medium and placed in a Petri dish with ½ MS agar. Petri dishes were closed with parafilm (Bemis) and were placed in a M-5-Z growth cabinet (PolyKlima) with a 11/13 day/night interval (including 30 min dusk and 30 min dawn during which the lights slowly increase in intensity), 80% relative humidity, and 210 μmol s^−1^m^−2^ light intensity.

Gnotobiotic culture boxes were prepared following the methods described by [48]. In brief, Magenta Culture Boxes GA-7 were filled with 90-g-fine zeolite clay granulate (Klinoptilolith, 0.2–0.5 mm, Labradorit.de) and autoclaved with lids closed. Lids were previously perforated and holes were covered with a double layer of gas permeable tape (Micropore, 3M). After autoclaving and cooling down, 45 ml of sterile ¾ MS Medium was added per box under aseptic conditions. One week after sowing, seedlings were transferred, including the tips they were germinated on, into prepared Magenta boxes. Four seedlings were placed per box and grown under the same conditions described above.

### Plant Inoculation

To prepare the inoculum of the different strains, a single colony was selected and used to prepare overnight cultures in LB medium (HiMedia, Pe299R_CUSPER_ with 50 mg L^−1^ kanamycin, respectively). The overnight cultures were then used to inoculate fresh liquid cultures by adding 500 µl of cultures to 50 ml fresh medium. Those cultures were grown to mid exponential phase while shaking at 30 °C and 200 rpm. Pe299R_CUSPER_ cultures were supplemented with kanamycin and 1 mM isopropylthio-β-galactoside (IPTG) to induce GFP expression [38]. When reaching log-phase after approximately 6 h of growth, cells were pelleted by 5 min of centrifugation at 4000 ✕ *g*, washed three times, and adjusted to an OD600nm of 0.1 with sterile phosphate buffered saline (PBS, 1.37 M NaCl, 27 mM KCl, 100 mM Na_2_HPO_4_, 18 mM KH_2_PO_4_, pH 7). Where appropriate, strains were mixed prior to inoculation, resulting in a relative OD600nm = 0.05 for each strain.

To inoculate *A. thaliana* plants, Magenta® box lids were temporarily replaced with a lid with only one central hole. Each box was then sprayed with 200 µl of inoculum with an airbrush paint gun (Ultra Spray gun, Harder & Steenbeck, Norderstedt, Germany). Then, the lids were replaced and plants were placed back into the growth chamber.

### Plant Sampling and Bacterial Cell Recovery

Three-week-old plants were sampled after 0 h, 12 h, 18 h, and 24 h post inoculation. Plants were sampled by harvesting the total aboveground material using sterile forceps and scissors. Plant material was transferred into a 15-ml centrifuge tube, and fresh weight was determined before 1 ml PBS was added to recover leaf surface-attached bacteria. The samples were then vortexed for 15 s and sonicated for 5 min at 75% intensity in a sonication bath (Emmi 12 HC, EMAG). Leaf washes were recovered from the tubes and a 100 µl aliquot was used to determine colony-forming units (CFU) by serial dilution on LB agar supplemented with rifampicin (50 mg/L) and kanamycin (50 mg/L) to select for Pe299R_CUSPER_. The remaining leaf wash was centrifuged for 10 min at 15,000 × *g* at 4 °C, the supernatant was discarded, and the resulting bacterial pellet resuspended was fixed overnight in 4% w/v paraformaldehyde in PBS at 4 °C. After fixation, the PFA was removed by three washing steps with sterile PBS. After the last washing step, the pellets were resuspended in 50 µl PBS mixed with 50 µl 96% v/v ethanol and stored at − 20 °C until they were analyzed. All samples were analyzed within 2 weeks.

### Microscopical Analysis of CUSPER Signals and Image Cytometry

Recovered bacterial cells were analyzed using widefield fluorescence microscopy as described elsewhere [11]. Briefly, cells were drop spotted onto 1% w/v agarose slabs on a microscopy slide and covered with a cover slip. An AxioImager Z2 microscope (Zeiss) with an Axiocam 712 mono camera (Zeiss) and X-cite Xylis broad spectrum LED light source (Excelitas) were used. Images were acquired at 1000 × magnification (EC Plan-Neofluar 100 × /1.30 Ph3 Oil M27 objective) in phase contrast, green fluorescence, and red fluorescence (Zeiss filter sets 38HE (BP 470/40-FT 495-BP 525/50) and 43HE (BP 550/25-FT 570-BP 605/70), respectively) using the software Zen 3.3 (Zeiss). At least 120 cells were acquired per biological replicate which consisted of bacteria pooled from four different plants. Images were analyzed using FIJI and as described previously [11, 49]. Briefly, Pe299_CUSPER_ cells were identified using their constitutive red fluorescence and the thresholding method “intermodes.” The resulting regions of interest were converted into a binary mask. Artifacts were excluded by analyzing only particle sizes from 0.5 to 2.5 µm and excluding cells touching the image edges. All images were manually curated using the phase contrast images to exclude false positive red fluorescent particles. The mask was then used to determine green fluorescence intensity of Pe299R_CUSPER_ cells. In addition, the average background fluorescence was measured by randomly sampling the background area of each image. Background fluorescence was subtracted from the data. As previously described, the number of experienced divisions of every cell was determined by calculating log_2_(average cell’s GFP fluorescence at *t* = 0 divided by single cell’s fluorescence at time *t*) [38].

## Results

### Pseudomonads Negatively Affect Growth of Pe299RCUSPERIn Vitro

**Fig. 1:**
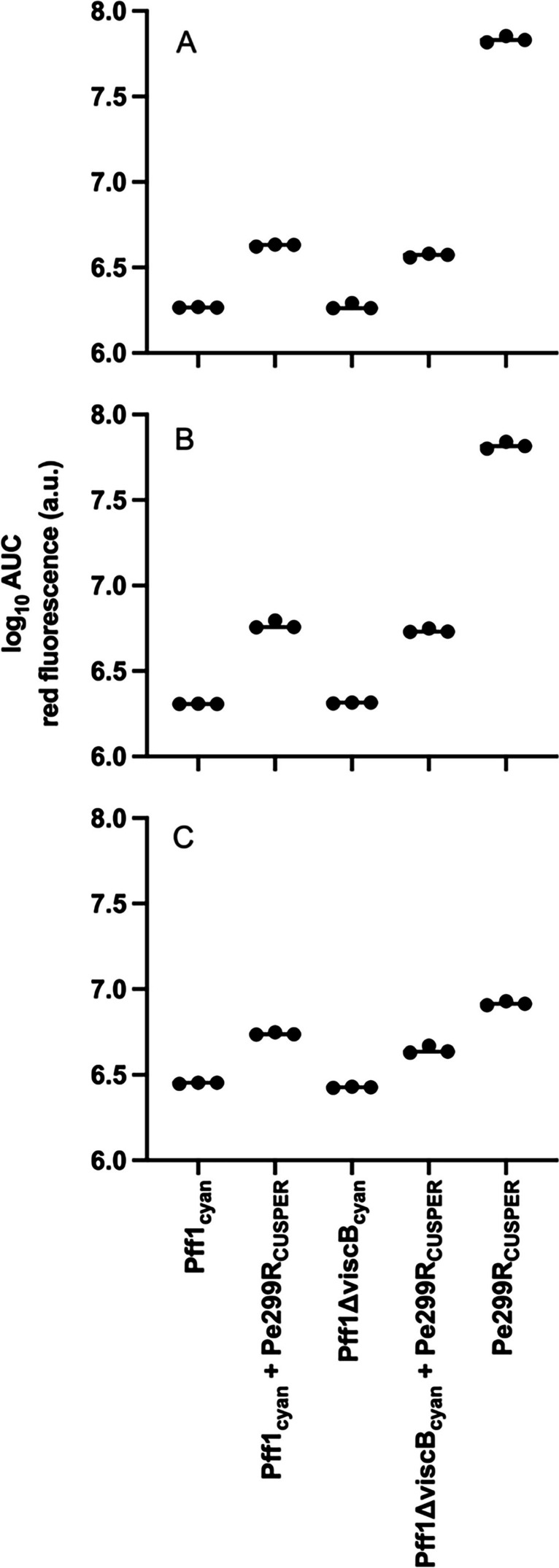
Impact of Pff1cyan and Pff1ΔviscB_cyan_ on the growth of Pe299R_CUSPER_ in different media. The effect on Pe299R_CUSPER_ growth was measured by determining the area under the curve of the red fluorescence of Pe299R_CUSPER_ monocultures and co-inoculations of Pe299R_CUSPER_ and the pseudomonads. **A**) M9 supplemented with glucose **B**) M9 supplemented with glucose, fructose and sorbitol and **C**) LB. Note that the y-axis is on a log10 scale

To investigate the interaction of Pe299R_CUSPER_ with Pff1_cyan_ or Pff1ΔviscB_cyan_ in homogeneous conditions, they were grown in shaken liquid cultures. Under these conditions, there was a strong decrease of Pe299R_CUSPER_ red fluorescence when it was co-inoculated with Pff1_cyan_ or Pff1ΔviscB_cyan_ (Fig. 1). Different media had slightly different effects on this interaction, and the decrease ranged from > 90% decrease in M9gluc or M9 3C to > 30% reduction in LB. This effect was not associated with the *Pseudomonas* red autofluorescence. By contrast, the effect of Pe299R_CUSPER_ on Pff1_cyan_ and Pff1ΔviscB_cyan_ was much smaller. Only in rich LB growth medium, we could detect a significant negative effect of Pe299R_CUSPER_ on the pseudomonads (Supplemental Fig. 1). In conclusion, the pseudomonads had a strong negative effect on Pe299R_CUSPER_ in vitro, while Pe299R_CUSPER_ is barely affecting the growth of the pseudomonad strains.

### Swarming on Solid Media

**Fig. 2:**
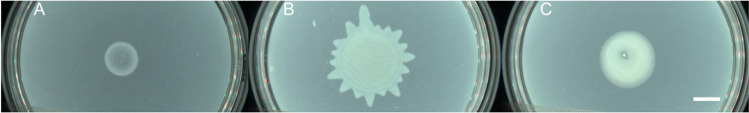
Colony morphology of **A** Pe299R_CUSPER_, **B** Pff1_cyan_, and **C** Pff1ΔviscB_cyan_ on KB swarming agar. Pictures were taken in a darkfield illuminator after 24 h of growth at 30 °C. Scale bar = 1 cm

When inoculated onto soft KB agar medium alone, the different strains showed various swarming patterns. Pe299R_CUSPER_ alone formed as a colony with entire edges that is slightly more slimy compared to growth on standard agar media and does not grow beyond the initial location of inoculation. Pff1_cyan_ grew in a flower-shaped colony that is indicative for swarming, far beyond the initial site of inoculation. By contrast, Pff1ΔviscB_cyan_ formed a colony similar to Pe299R_CUSPER_ and was not able to move far beyond the initial site of inoculation (Fig. 2).

**Fig. 3:**
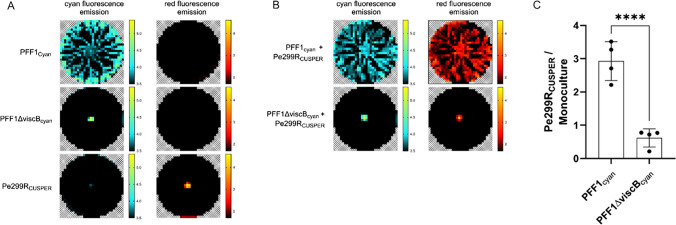
Spatially resolved analysis of bacterial growth and swarming behavior on LB soft agar plates after 16 h. **A** Monocultures of Pe299R_CUSPER_, Pff1_cyan_, and Pff1ΔviscB_cyan_ or **B** mixed cultures of Pe299R_CUSPER_ in combination with Pff1_cyan_ or Pff1ΔviscB_cyan_ on LB soft agar plates. Bacterial growth and biomass were tracked with a fluorescent microtiter plate reader. Growth of Pe299R_CUSPER_ was determined by measuring red fluorescence emission, and growth of pseudomonads was determined by measuring cyan fluorescence emission. Note that the color scales are presented as the decadic logarithm of arbitrary fluorescence units. Furthermore, the experiment was repeated four times on LB soft agar plates. **C** The fluorescence of Pe299R_CUSPER_ was determined after 16 h, and the ratio of Pe299_CUSPER_ growing in mixtures over its monoculture was determined

To investigate bacterial behavior after co-inoculating Pe299R_CUSPER_ with the two different pseudomonads, we used their respective constitutively expressed fluorescent proteins to track their biomass on a swarming agar (Fig. 3). While monocultures behaved as expected (Fig. 3A), we found that co-inoculated bacteria affected each other in different fashions. In co-culture with the swarming Pff1_cyan_, Pe299R_CUSPER_ had a much wider distribution on the agar surface (Fig. 3B). By contrast, in co-culture with the non-swarming Pff1ΔviscB_cyan_, Pe299R_CUSPER_ was similarly restricted in its distribution as in a monoculture. By repeating the experiment four times, we were able to determine the total change in biomass during mono and co-cultures (Fig. 3C). As a result, the Pe299R_CUSPER_ biomass, as measured by red fluorescence, was significantly higher in co-inoculations with surfactant-producing Pff1_cyan_ compared to Pe299R_CUSPER_ monocultures and co-inoculations with non-surfactant-producing Pff1ΔviscB_cyan_ (*p* = 0.0001 and < 0.0001, respectively, one-way ANOVA Tukey’s multiple comparison test). By contrast, albeit not significant, Pe299R_CUSPER_ biomass is slightly reduced during co-culture with Pff1ΔviscB_cyan_.

### Co-inoculation In Planta Does Not Significantly Affect Pe299R_CUSPER_ at the Population Scale but Does Affect Reproductive Success at the Single-Cell Resolution

To investigate the effect of a co-colonizer-producing surfactant on Pe299R_CUSPER_ in planta, the strains were co-inoculated onto axenic *A. thaliana* plants. The two different co-colonizing pseudomonads affected the population size of Pe299R_CUSPER_ similarly. Although we observed a trend that the population was slightly higher after 24 h in one of the experiments, this was not consistent between experiments (Supplemental Fig. 2).

**Fig. 4:**
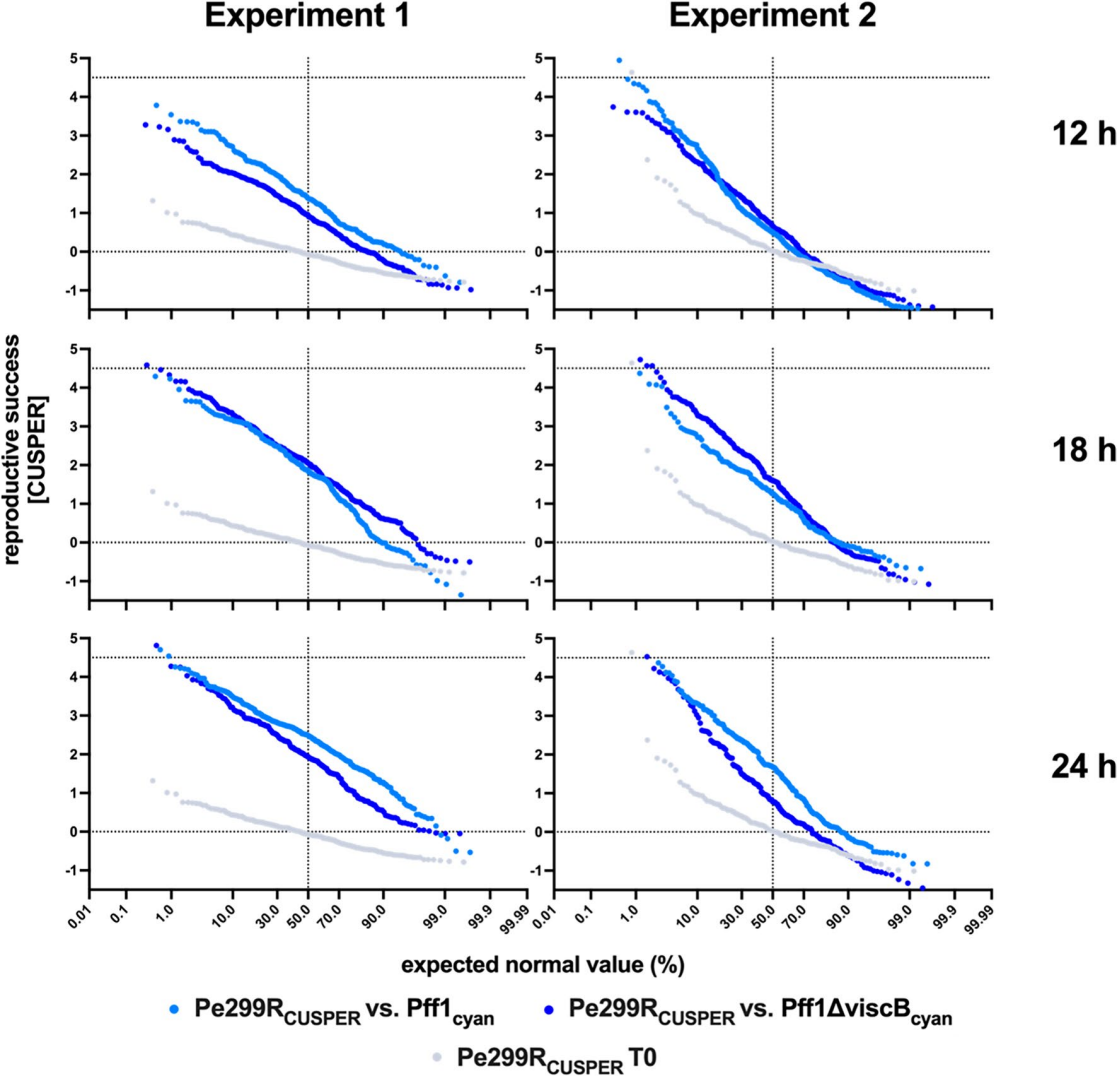
Reproductive success of individual Pe299R_CUSPER_ cells during co-colonization of leaves with Pff1_cyan_ or Pff1ΔviscB_cyan_. In gray, the respective T0 fluorescence intensity of the Pe299R_CUSPER_ population is depicted. Every increase in reproductive success depicts a cell division relative to the T0 population. Every sample is pooled from the bacteria recovered from four plants

By contrast, at the single-cell resolution, there are noteworthy differences in the population development during the co-colonization of leaves (Fig. 4). Generally, a proportionally larger subpopulation of Pe299R_CUSPER_ experienced more cell divisions in the presence of the surfactant-producing Pff1_cyan_ compared to the non-producing Pff1ΔviscB_cyan_. This effect is most apparent after 24 h and was reproduced in three independent experiments (Fig. 4 and Supplemental Fig. 3). Furthermore, we observed that after 18 h, the presence of the non-surfactant-producing strain had a positive effect on Pe299R_CUSPER_ (Fig. 4).

## Discussion

The potential roles of biosurfactants in plant–microbe interactions have been investigated from many different angles: their hygroscopic nature enhances the survival of pseudomonads [27] and increases water availability on leaves [28], they are suspected to increase diffusion of nutrients through the hydrophobic leaf cuticle [31], and they have been shown to increase alkane degradation by leaf-colonizing bacteria [29]. Generally, the abundance of biosurfactant-producing bacteria on leaves is proportionally high [30, 31, 50], indicating a potential ecological importance during life on leaves. Here, we show that surfactant-producing bacteria may facilitate the growth of co-colonizers on leaves in a surfactant production and surface colonization dependent manner.

Being both copiotrophic generalists that exhibit a wide range of nutrient utilization, it was expected that Pe299R_CUSPER_ and both pseudomonads affected each other strongly in liquid culture. Indeed, it was mostly Pe299R_CUSPER_ that was negatively affected in minimal media and less so in complex LB medium. In turn, the pseudomonads were almost not affected at all by the presence of Pe299R_CUSPER_ in minimal media and to a smaller degree in LB medium. These results are in stark contrast to our observations on a spatially structured soft agar surface. Here, the non-surfactant-producing Pff1ΔviscB_cyan_ negatively affected Pe299R_CUSPER_, but not Pff1_cyan_ which instead increased the amount of Pe299R_CUSPER_ fluorescence as a proxy for biomass by a factor of three and thereby increased its fitness dramatically. As this increase correlates to a larger spread of the Pe299R_CUSPER_, it is likely that this increase can be accredited to a mobilization of Pe299R_CUSPER_ by Pff1_cyan_, similar co-swarming phenomena have previously been observed in *Paenibacillus vortex* and other *Paenibacillus* strains [51], as well as *Pseudomonas aeruginosa* and *Burkholderia cenocepacia* [32, 52]. Generally, it has been shown that the swarming conferring strain gains a benefit from the co-swarmer by for instance taking advantage of antibiotic resistances provided by the immobile strain [51], or by recovering mobility in case one strain loses mobility factors [32, 52]. In our study, there is no apparent benefit for the strains that mobilize Pe299R_CUSPER_. Instead, it is Pe299R_CUSPER_ that seems to be able to take advantage of the swarming strains and increases its fitness as compared to homogeneous shaken liquid cultures where swarming does not confer any fitness advantages. This observation led us to test if this effect also pertains to colonization of leaf surfaces, which is the origin of isolation of all strains used in this study.

**Fig. 5:**
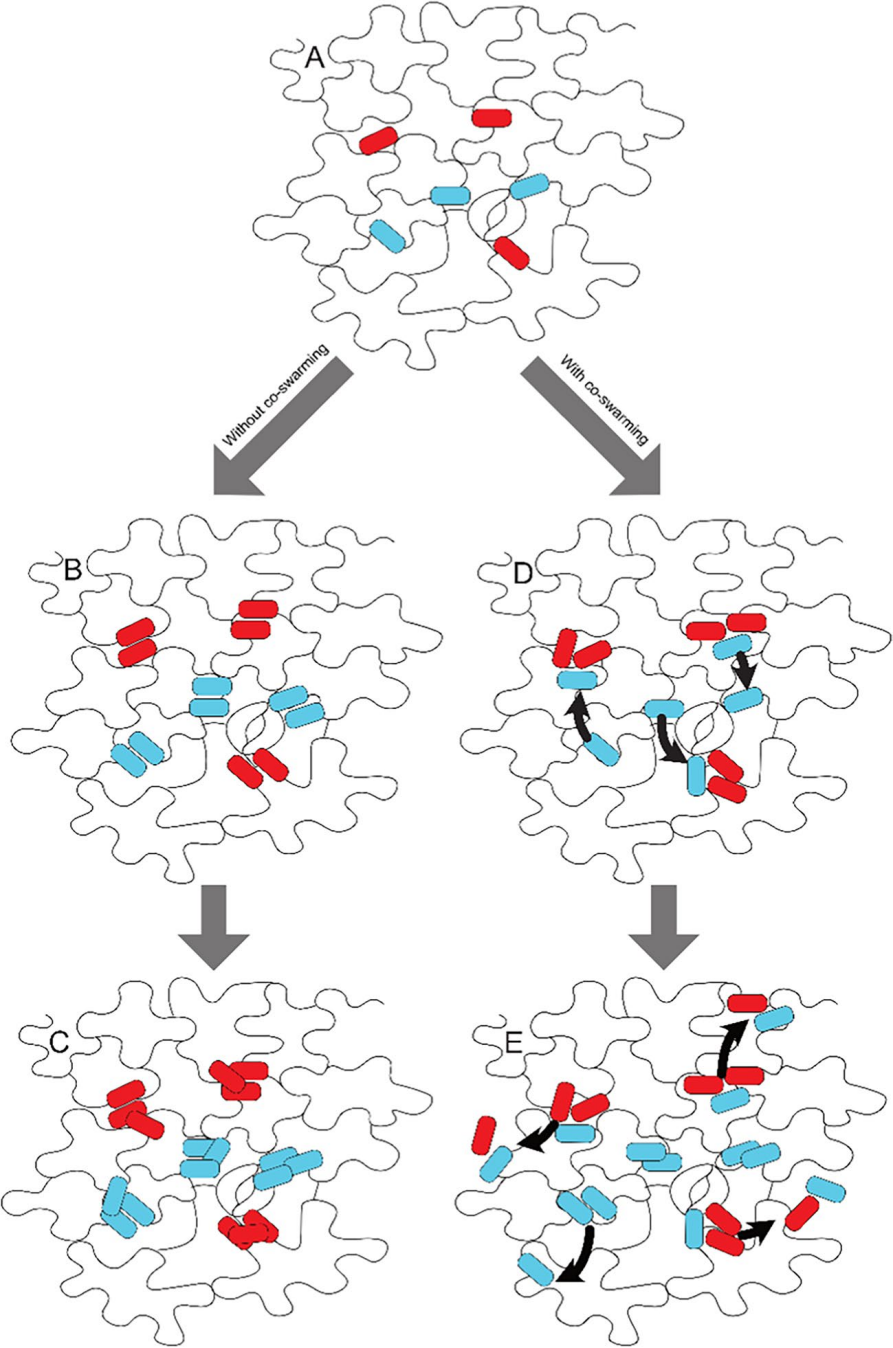
Model explaining behavior of Pe299_CUSPER_ after co-inoculation with Pff1ΔviscB_cyan_ (**B**, **C**) or Pff1cyan (**D**, **E**). (**A**) The initial distribution of non-motile, red-colored Pe299_CUSPER_ cells and co-inoculated cyan-colored Pff1ΔviscB_cyan_ or Pff1_cyan_ cells. In the left scenario, cyan cells do not produce any surfactants and do not swarm. As a result red and cyan populations remain localized and rely on the locally available nutrients. In the right scenario, cyan cells produce surfactants and swarm. This leads to a higher probability of cyan cells to encounter red cells, which leads to a temporally high competition for nutrients and a reduction of red cell divisions (**D**). However, if red cells are mobilized by co-swarming as a result of close spatial proximity to cyan cells, this leads to the exploration of new sites and an increase of growth

To test this, we tracked the changes of Pe299R_CUSPER_ populations on *A. thaliana* and the ability of Pe299R_CUSPER_ individual cells to divide on leaves, exploiting the reproductive success bioreporter CUSPER. At the population scale, the effect of co-colonization was minimal, although a trend of higher Pe299R_CUSPER_ populations during co-colonization with Pff1_cyan_ as compared to co-colonization with Pff1ΔviscB_cyan_ could be observed (Supplemental Fig. 2). This is in contrast to single-cell observations, where we could detect an increased number of cells that experienced a notably higher number of divisions in the presence of Pff1_cyan_ as compared to the presence of Pff1ΔviscB_cyan_ (Fig. 4) after 24 h. Curiously, this effect was not visible after 18 h where Pe299R_CUSPER_ seemed to have a minimal growth advantage in the presence of Pff1ΔviscB_cyan_. To explain this, we hypothesize the following model of interactions: During inoculation, it is more probable for individual strains to arrive on the leaf without a competitor in their local environment (Fig. 5(A)). In a scenario without swarming (Fig. 5(B) and (C)), the chance of bacterial strains interacting with each other is low as the minimal interaction distance between bacteria on leaves is about 10 µm [13, 53]. As a consequence, bacteria can initially grow rather unimpeded by competition. By contrast, in a scenario with swarming (Fig. 5(D) and (E)), the swarming strain will have higher chances to meet the non-swarming Pe299R_CUSPER_, which leads to high local competition and locally reduced populations of Pe299R_CUSPER_ (compare Fig. 3, where the local biomass of Pe299R_CUSPER_ is reduced during competition). However, as co-swarming leads to mobilization of Pe299R_CUSPER_ and allows the exploration of new microenvironments, this should lead to an increased reproductive success of the strain despite the local competition (Fig. 5(E)). This is directly analogous to the scenario observed on the spatially structured agar surface (Fig. 3).

### Effect of Other Surfactant-Producing Pseudomonads on Pe299R_CUSPER_

Interestingly, in a previous study, we have observed a similar effect of increased reproductive success of Pe299R_CUSPER_ when co-colonizing plant leaves with surfactant-producing *P.* *koreensis* P19E3 and *P. syringae* B728a despite high overlap in resource utilization abilities [11]. This effect can now likely be accredited to the production of surfactants and co-swarming. To further test if co-swarming of Pe299R_CUSPER_ with pseudomonads can be generalized and accredited to surfactant production, we have tested the growth Pe299R_CUSPER_ and three additional pseudomonads and their respective surfactant knockout mutants on soft agar. Indeed, we could observe that the *Pseudomonas* strains Pff2 and Pff4, but not their respective surfactant knockout mutants to co-swarming of Pe299R_CUSPER_ (Supplemental Fig. 4). Intriguingly, Pff3 was not able to mobilize Pe299R_CUSPER_. Pff3 also exhibited a different style of swarming on soft agar plates which seemed to consist of a very thin layer of biomass (Supplemental Fig. 5), which might explain this difference in co-swarming. Exploring the reasons for those differences is beyond the scope of this study and might be growth medium dependent as well as strain dependent as bacteria have been shown to be diverse in their swarming behavior [54, 55].

## Conclusion

While pseudomonads act as strong competitors in homogeneous environments, in spatially structured environments, they affect co-colonizing *P. eucalypti* 299R positively. While this effect cannot be observed on a population scale during leaf colonization, we provide evidence that *P. eucalypti* 299R takes advantage of the presence of surfactant-producing pseudomonads during leaf colonization. This stresses the importance of surfactants produced by pseudomonads as public goods during leaf colonization and implies that cheating of *P. eucalypti* 299R and possibly other taxa may be the rule rather than the exception. Our study highlights another pivotal role of surfactants in the phyllosphere and the implications of surfactant production in this environment. Particularly in the context of preparing microbial inocula for the application on plants, our findings provide additional traits, i.e., surfactant production and the ability for co-swarming, which should be considered during the formulation of products [].

### Supplementary Information

Below is the link to the electronic supplementary material.

Supplementary file1 (DOCX 5231 KB)

## Data Availability

The raw data of this article is available at 10.5281/zenodo.10610551.
