# Supplementary material for: Hitching a Ride in the Phyllosphere: Surfactant Production of Pseudomonas spp. Causes Co-swarming of Pantoea eucalypti 299R
Source: Microb Ecol. 2024 Apr 29;87:62. doi: 10.1007/s00248-024-02381-4 (PMC11058625; doi:10.1007/s00248-024-02381-4)
Supplement: Supplementary file 1 [file 248_2024_2381_MOESM1_ESM.docx]

**Hitching a ride in the phyllosphere: Surfactant production of *Pseudomonas* spp. causes co-swarming of *Pantoea eucalypti* 299R**

Michael Kunzler^1^, Rudolf O. Schlechter^1^, Lukas Schreiber^2^ and Mitja N.P. Remus-Emsermann^1^

^1^Institute for Biology - Microbiology, Freie Universität Berlin, Königin-Luise Straße 12-16, 14195 Berlin, Germany

^2^Institute for Cellular and Molecular Botany, Bonn University, Kirschallee 1-3, 53115 Bonn, Germany

* Correspondence: [m.remus-emsermann@fu-berlin.de](mailto:m.remus-emsermann@fu-berlin.de)

**Supplemental data**

**Supplemental Figure 1** Impact of Pe299R_CUSPER_ on growth of Pff1_cyan_ and Pff1ΔviscB_cyan_ in different media: A) M9 supplemented with glucose B) M9 supplemented with glucose, fructose and sorbitol and C) LB. Cyan fluorescence of Pe299R_CUSPER_ Pff1_cyan_ and Pff1ΔviscB_cyan_ was tracked over time and the area under the curve was determined. Only the decrease of the cyan fluorescence signal during co-growth of Pe299R_CUSPER_ and the pseudomonads in rich medium is significantly different from the respective monocultures (unpaired t-test, P < 0.001).

**Supplemental Figure 2** Growth of Pe299R_CUSPER_ co-cultured with Pff1_cyan_ and Pff1ΔviscB_cyan_ *in planta*. No significant differences were detected on the population scale. For every treatment, four plants were harvested per time point, the experiment was performed three times independently. Two independent experiments are shown.

**Supplemental Figure 3** Reproductive success of individual Pe299R_CUSPER_ cells during co-colonization of leaves with Pff1_cyan_ or Pff1ΔviscB_cyan_, third independent experiment. In gray, the respective T0 fluorescence intensity of the Pe299R_CUSPER_ population is depicted. Every increase in reproductive success depicts a cell division relative to the T0 population. Every sample is pooled from the phyllosphere material of four individual plants..

**Supplemental Figure 4** Pe299R_CUSPER_ co-colonizing soft LB agar with Pff2, Pff3, Pff4 and their respective surfactant knockout mutants measured using a fluorescent plate reader (lower line). RFP emission depicts the distribution of Pe299R_CUSPER_.

**Supplemental Figure 5** Macroscopical images of Pff2, Pff3, Pff4 and their knockout mutants on soft LB agar after 12 hours of growth. A-C) Wild type strains. D-F) Knockout mutants. Scale bar = 1 cm.
